# Activity Change in Response to Bad Air Quality, National Health and Nutrition Examination Survey, 2007–2010

**DOI:** 10.1371/journal.pone.0050526

**Published:** 2012-11-30

**Authors:** Ellen M. Wells, Dorr G. Dearborn, Leila W. Jackson

**Affiliations:** 1 Department of Environmental Health Sciences, Case Western Reserve University School of Medicine, Cleveland, Ohio, United States of America; 2 Department of Epidemiology and Biostatistics, Case Western Reserve University School of Medicine, Cleveland, Ohio, United States of America; University of Pittsburgh, United States of America

## Abstract

Air pollution contributes to poor respiratory and cardiovascular health. Susceptible individuals may be advised to mitigate effects of air pollution through actions such as reducing outdoor physical activity on days with high pollution. Our analysis identifies the extent to which susceptible individuals changed activities due to bad air quality. This cross-sectional study included 10,898 adults from the National Health and Nutrition Examination Survey (NHANES) 2007–2010. Participants reported if they did something differently when air quality was bad. Susceptible categories included respiratory conditions, cardiovascular conditions and older age (≥65 years). Analyses accounted for complex survey design; logistic regression models controlled for gender, race, education, smoking, and body mass index. 1305 individuals reported doing something differently (12.0%, 95% confidence interval (CI): 10.9, 13.1). This percentage was 14.2% (95% CI: 11.6, 16.8), 25.1% (95% CI: 21.7, 28.6), and 15.5% (95% CI: 12.2, 18.9) among older adults, those with a respiratory condition, and those with a cardiovascular condition, respectively. In adjusted regression models the following were significantly more likely to have changed activity compared to those who did not belong to any susceptible group: respiratory conditions (adjusted odds ratio (aOR): 2.61, 95% CI: 2.03, 3.35); respiratory and cardiovascular conditions (aOR: 4.36, 95% CI: 2.47, 7.69); respiratory conditions and older age (aOR: 3.83; 95% CI: 2.47, 5.96); or all three groups (aOR: 3.52; 95% CI: (2.33, 5.32). Having cardiovascular conditions alone was not statistically significant. Some individuals, especially those with a respiratory condition, reported changing activities due to poor air quality. However, efforts should continue to educate the public about air quality and health.

## Introduction

Ample evidence links ambient air pollution with decreased health outcomes. Fine particulate matter and ozone have been linked with increased respiratory and cardiovascular morbidity and mortality [1]–[3]. Air pollution exposure is a major public health concern: using air quality data from 2005, Fann et al. estimated 130,000 deaths in the United States were related to particulate matter exposure and 4700 deaths were related to ozone exposure [4].

Moreover, reductions in these pollutants have been shown to reduce rates of related conditions [5], [6]. Reduced exposure to air pollutants may occur regionally via reduced emissions; however, individuals may also reduce their personal exposures through actions such as shortening the duration and intensity of outdoor activities on days with elevated air pollution. For example, Langrish et al. showed in an open randomized case-crossover trial that wearing a mask while walking outdoors was associated with improved cardiovascular function among patients with coronary heart disease [7].

The United States Environmental Protection Agency (US EPA) recommends that individuals susceptible to the health effects of air pollution take precautions at lower concentrations than the general population, as susceptible individuals may be affected at these concentrations [8]. Susceptible populations include infants and children, the elderly, and those with respiratory or cardiovascular health conditions [9]–[13]. Physicians are also urged to encourage their patients, especially those in susceptible groups, to limit their exposure to ambient air on days with elevated air pollution [14]–[17].

The objective of this study was to determine the extent to which individuals take action to limit their exposure to ambient air pollution based on knowledge of poor air quality, and whether those in a susceptible population were more likely to do so. Susceptible groups considered in the present analysis include those with a self-reported respiratory condition (asthma, emphysema, and chronic bronchitis), those with a self-reported cardiovascular condition (congestive heart failure, coronary heart disease, angina, heart attack or stroke) and the elderly (≥65 years of age).

## Materials and Methods

### Study Population

This is a cross-sectional study using data from the 2007–2010 National Health and Nutrition Examination Survey (NHANES) conducted by the National Center for Health Statistics (NCHS) within the United States Centers for Disease Control and Prevention (CDC) [18]. Additional details about NHANES are available at http://www.cdc.gov/nchs/nhanes.htm. Participants provided written informed consent, and the study was operated under approval from the NCHS Research Ethics Review Board. This analysis had approval from the Case Western Reserve University Institutional Review Board.

Inclusion criteria included participation in both the household interview and the health examination for NHANES 2007–2010 (n = 20,015). Those less than 20 years of age (n = 8249) were excluded because key self-reported medical conditions were only asked among those 20 years or older. Other individuals were excluded due to missing data on key variables including activity change due to air quality (n = 2); educational status (n = 20); body mass index (BMI) (n = 159); or blood cotinine (n = 687). A total of 10,898 individuals were included in analyses.

### Activity Change

Within the air quality questionnaire, activity change was determined from the question “During the past 12 months, when you thought or were informed air quality was bad, did you do anything differently?” [18]. Respondents could answer “yes”, “no” or “never thought/not informed about air quality”; hereafter referred to as “not informed” for brevity. For analyses, those in the not informed category were included with those who said no, as these individuals did not change their activities based upon knowledge about air quality. The supporting information includes a sensitivity analysis, where models were also constructed when excluding individuals in the not informed category.

Individuals who responded yes to the air quality question were then asked if they made any of the following changes: wore a mask; spent less time outdoors; avoided roads that have heavy traffic; did less strenuous activities; took medication; closed windows of your house; drove your car less; canceled outdoor activities; exercised indoors instead of outside; used buses, trains or subways; or other. During data editing, a new category, “used or changed air filter or air cleaner”, was created for responses that mentioned doing so. In a second sensitivity analysis, we created additional models where activity change was limited to those who identified making changes which could have resulted in reducing their exposure to ambient air pollution or reducing the severity of health effects resulting from their exposure. These included activities which may reduce exposure (spent less time outdoors, closed windows of your house, canceled outdoor activities, exercised indoors instead of outside, wore a mask, did less strenuous activities) and activities which may reduce the impact of exposure (took medication).

### Susceptible Groups

Three groups susceptible to the health effects of air pollution were considered in analyses: 1) the elderly; 2) those with a respiratory condition; and 3) those with a cardiovascular condition. The elderly were defined as those 65 years of age or older. Respiratory condition was based on self-report of at least one of the following from the medical conditions questionnaire: current asthma, emphysema, or current bronchitis. Similarly, cardiovascular condition was based on self-report of at least one of the following from the medical conditions questionnaire: congestive heart failure, coronary heart disease, angina, heart attack or stroke. As there is substantial overlap among these three variables, we created a susceptible category variable which classified participants according to their status for each of the three susceptible groups.

### Additional Covariates

The demographics questionnaire provided data on age, education level and race/ethnicity. Age was categorized into those 20–34, 35–49, 50–64 and 65 and older for descriptive statistics. Education was categorized as less than high school, high school, some college or a 2-year degree, and a 4-year degree or higher. Race/ethnicity was categorized as Hispanic, non-Hispanic white, non-Hispanic black, and other or mixed race.

Serum cotinine levels were measured as an indicator of smoking status. Whole blood specimens were collected by trained medical staff and analyzed for cotinine using dilution-high performance liquid chromatography/atmospheric pressure chemical ionization tandem mass spectrometry. Limit of detection was 0.015 ng/mL. Smoking status was based on serum cotinine levels, where <1 ng/mL indicates a nonsmoker, 1–10 ng/mL indicates a passive smoker and >10 ng/mL indicates an active smoker [19].

Height and weight were was obtained by trained health technicians during the health examination and used to calculate BMI (kg/m^2^). BMI was categorized using standard definitions (<18.5 kg/m^2^ =  underweight; 18.5–24.9 kg/m^2^ = normal weight; 25–29.9 kg/m^2^ =  overweight and ≥30 kg/m^2^ = obese) [20]; the underweight category was then combined with the normal weight category due to the small number of underweight individuals (population weighted proportion: 1.5%).

### Data Analysis

All data were analyzed using Stata 11.2 (College Station, TX). Appropriate survey weights were used to account for the complex design and non-response. Sampling error was estimated using the Taylor series linearized method. Reported sample sizes are those of the study; however, proportions, percentages and odds ratios are all population-based estimates.

Descriptive statistics were used to assess the frequencies and distribution of individual variables, particularly in relationship to activity change and susceptible groups. Pearson’s chi-square test was used to evaluate differences across groups. The association between activity change (dependent variable) and susceptibility category (independent variable) was created using logistic regression models. Covariates were considered for inclusion in the model based on *a priori* hypotheses and strength of the relationship with activity change in bivariate analyses. The final logistic regression model was adjusted for gender, educational level, race/ethnicity, smoking and body mass index.

## Results

Population demographics are presented in Table 1. Mean age of the study population was 46.9 years (95% confidence interval (CI): 46.3, 47.6); 17.0% (95% CI: 15.9, 18.1) were at least 65 years old. Slightly more than half of the participants were women (51.8%, 95% CI: 50.9, 52.6), and the majority (69.3%, 95% CI: 64.3, 74.4) were non-Hispanic white. Mean body mass index was 28.6 kg/m^2^ (95% CI: 28.4, 28.8). Serum cotinine levels were highly right skewed, with a geometric mean of 0.36 ng/mL (95% CI: 0.30, 0.44). The range of cotinine was from below the limit of detection to 1438 ng/mL. Active smokers comprised 25.8% (95% CI: 23.9, 27.6) of the population.

**Table 1:** Population characteristics by activity change status, NHANES 2007–2010, N = 10,898.

		Changed activity		Did not change activity		Not informed about air quality	
Characteristic		N^a^	Percent (95% CI)^b^	N^a^	Percent (95% CI)^b^	N^a^	Percent (95% CI)^b^
Entire population		1305	12.0 (10.9, 13.1)	8895	81.5 (79.5, 83.5)	698	6.5 (4.5, 8.6)
Age^c^	20–34 years	230	8.3 (6.6, 9.9)	2244	85.6 (85.6, 88.6)	2635	6.2 (4.0, 8.3)
	35–49 years	336	12.3 (10.5, 14.0)	2329	80.9 (78.2, 83.6)	2845	6.8 (4.4, 9.3)
	50–64 years	398	14.2 (12.0, 16.4)	2186	79.7 (76.7, 82.6)	2752	6.1 (3.7, 8.5)
	≥65 years	341	14.2 (11.6, 16.8)	2136	79.7 (76.5, 82.6)	2666	7.0 (4.3, 9.8)
Gender^c^	Male	513	9.3 (8.2, 10.5)	4453	84.4 (82.2, 86.7)	335	6.3 (4.1, 8.5)
	Female	792	14.5(13.0, 15.9)	4442	78.8 (76.6, 80.9)	363	6.7 (4.7, 8.8)
Education^c^	Less than high school	243	7.9 (6.5, 9.3)	2737	84.4 (81.8, 87.0)	246	7.8 (4.8, 10.7)
	High school	281	10.6 (8.8, 12.4)	2181	84.5 (82.7, 86.4)	124	4.9 (3.4, 6.4)
	Some college or 2-year degree	436	13.3 (11.7, 15.0)	2315	79.5 (82.7, 86.4)	188	7.2 (4.6, 9.9)
	4-year college degree or higher	345	14.8 (12.1, 17.5)	1662	78.9 (75.9, 81.9)	140	6.3 (4.0, 8.6)
Race/ethnicity^c^	Non-Hipanic white	659	12.3 (10.8, 13.7)	4222	80.9 (78.6, 83.2)	367	6.9 (4.4, 9.3)
	Non-Hispanic black	293	14.2 (10.4, 17.9)	1637	83.8 (79.7, 87.9)	43	2.0 (1.2, 2.8)
	Hispanic	276	8.0 (6.0, 10.0)	2616	83.8 (79.5, 88.0)	249	8.2 (4.2, 12.2)
	Other/mixed	77	14.0 (10.3, 17.7)	420	79.5 (74.0, 85.0)	39	6.5 (3.1, 9.9)
Serum cotinine^c^	<1 ng/mL	958	12.8 (11.5, 14.0)	6115	80.2 (78.1, 82.3)	538	7.1 (4.9, 9.2)
	1 to 10 ng/mL	50	10.2 (6.2, 14.2)	393	81.8 (76.5, 87.2)	30	8.0 (4.2, 11.7)
	>10 ng/mL	297	10.2 (8.7, 11.8)	2387	85.0 (82.7, 87.2)	130	4.8 (2.9, 6.8)
Body mass index	<25 kg/m^2^	354	11.5 (10.1, 12.8)	2552	82.1 (79.1, 85.0)	193	6.5 (4.2, 8.7)
	25–29 kg/m^2^	414	11.6 (9.9, 13.3)	3083	81.6 (79.6, 83.7)	247	6.8 (4.7, 8.9)
	≥30 kg/m^2^	537	12.9 (11.2, 14.6)	3260	80.8 (78.5, 83.2)	258	6.3 (4.0, 8.6)
Respiratory condition^c^	No	1018	10.6 (9.6, 11.6)	8120	82.8 (80.7, 85.0)	637	6.6 (4.5, 8.7)
	Yes	287	25.1 (21.7, 28.6)	775	69.1 (65.1, 73.0)	61	5.8 (3.1, 8.5)
Cardiovascular condition^c^	No	1121	11.7 (10.6, 12.8)	7971	81.8 (79.6, 84.0)	618	6.5 (4.5, 8.6)
	Yes	184	15.5 (12.2, 18.9)	924	78.0 (74.5, 81.4)	80	6.5 (3.7, 9.3)

NHANES = National Health and Nutrition Examination Survey; CI = confidence interval.

a Unweighted sample N.

b Percents are corrected for survey design, are row percents, and may not sum to 100 due to rounding.

c Significant (*p*<0.05) Pearson’s chi-squared test corrected for survey design, comparing the characteristic to activity change.

Current asthma was more prevalent than emphysema or current bronchitis (7.4% versus 1.8% and 2.5%, respectively) and 9.7% (95% CI: 8.4%, 10.9%) had at least one respiratory condition. The proportion with cardiovascular conditions ranged from 2.0% (angina) to 3.3% (heart attack), with 8.1% (95% CI: 7.2, 9.1) having at least one cardiovascular condition. Additional data regarding health outcomes is included in Table S1.

A total of 1305 (12.0%, 95% CI: 10.9, 13.1) individuals responded that they did something differently due to bad air quality (Table 1). Among those who reported changing an activity, the most commonly reported activities changed were spending less time outdoors (69.4% of those who had changed an activity) and closing the windows of your house (25.5%) (Table S2).

In bivariate comparisons, those more likely to change activities were older, women, those with more education, those with a respiratory condition, and those with a cardiovascular condition. Hispanics and active smokers were less likely to have changed activities.

Twenty-seven percent of the population belongs to at least one of the three susceptible groups (Table 2). The most common susceptible categories were a) those only >65 years old (10.9%), b) those only with a respiratory condition (6.4%), and c) those ≥65 with a cardiovascular condition (3.8%); all other susceptible group categories contained less than two percent of the study population.

**Table 2:** Prevalence of individuals susceptible to health effects from air pollution, NHANES 2007–2010, N = 10,898.

Susceptible category	N^a^	Percent (95% CI)^a^
None	7135	73.0 (71.4, 74.6)
Respiratory only	642	6.4 (5.5, 7.2)
Cardiovascular only	319	2.6 (2.3, 2.9)
≥65 years only	1713	10.9 (10.1, 11.8)
Respiratory and cardiovascular	136	1.0 (0.7, 1.3)
Respiratory and ≥65 years	220	1.6 (1.3, 1.8)
Cardiovascular and ≥65 years	608	3.8 (3.3, 4.3)
All three groups	125	0.7 (0.5, 0.9)

NHANES = National Health and Nutrition Examination Survey; CI = confidence interval.

a N is the unweighted sample N; percents are corrected for survey design and may not sum to 100 due to rounding.

Logistic regression model results are presented in Table 3, Table S3, and Figure S1. In adjusted regression models, there were significantly increased odds of having changed activity for those with a respiratory condition, either alone or in combination with another susceptible condition. The strongest relationships were among those with a cardiovascular and respiratory condition (adjusted odds ratio (aOR): 4.36, 95% CI: 2.47, 7.69) and those ≥65 years old with a respiratory condition (aOR: 3.83, 95% CI: 2.47, 5.96). Results were similar in the sensitivity analyses exploring the relationship when excluding the population not informed about air quality or limiting those who changed activities to those who changed an activity which may reduce exposure or the impact of exposure (Tables S3, S4).

**Table 3:** Odds ratio (95% confidence interval)^a^ for changing activity due to poor air quality by susceptible category, NHANES 2007–2010, N = 10,898.

Susceptible category	Unadjusted	Adjusted^b^
None (referent)		
Respiratory only	2.64 (2.06, 3.37)	2.61 (2.03, 3.35)
Cardiovascular only	1.16 (0.76, 1.77)	1.33 (0.86, 2.04)
≥65 years only	1.20 (0.93, 1.54)	1.22 (0.95, 1.57)
Respiratory and cardiovascular	4.06 (2.31, 7.15)	4.36 (2.47, 7.69)
Respiratory and ≥65 years	3.64 (2.35, 5.64)	3.83 (2.47, 5.96)
Cardiovascular and ≥65 years	1.23 (0.78, 1.91)	1.38 (0.89, 2.13)
All three groups	2.80 (1.94, 4.04)	3.52 (2.33, 5.32)

NHANES = National Health and Nutrition Examination Survey.

a The model incorporates complex survey design and survey weights.

b Model adjusted for gender, education, race/ethnicity, smoking status (based on serum cotinine), and body mass index category.

## Discussion

This research demonstrates that some individuals, particularly those with respiratory disease, changed activities in response to bad air quality. Our results are consistent with the limited body of literature exploring activity change in response to poor air quality. Wen et al. looked at change in outdoor activity based on individual perception of air quality as well as awareness of medical alerts using data from the 2005 Behavioral Risk Factor Surveillance System [21]. They found that 12.0% of those without asthma and 25.6% of those with lifetime asthma changed activities based on personal perceptions of bad air quality. Semenza et al. report 10–15% of respondents changed activities due to poor air quality based on a telephone survey during July-September 2005 in Houston, Texas and Portland, Oregon [22]. In our analysis, 12.0% of the study population changed activities due to bad air quality, and 25% of those with a respiratory condition changed activities (Table 1).

There was no association between having a cardiovascular condition or being at least 65 years old, without also having a respiratory condition, and changing activities due to bad air quality. Although the cause for this cannot be definitively determined from this study, it is possible that individuals with a respiratory condition are more likely to understand the connection between ambient air pollution and their personal health, as decreased respiratory function may be easier to detect in comparison with decreased cardiovascular function. In a survey of five United Kingdom neighborhoods, Howel et al. found between 82–89% of respondents thought asthma was related to air pollution and 69–78% of respondents thought bronchitis was related to air pollution; additionally, having the condition in question meant a person was more likely to perceive air pollution as affecting it [23]. In contrast, in a survey among patients of a cardiology outpatient clinic in Michigan, only 43% were aware that air pollution negatively affects the heart, and only 8.2% of patients had ever discussed health risks from outdoor air pollution with their doctors [24]. Wen and colleagues noted that advice from a professional, such as a physician, had an impact on the percentage of individuals changing activity [21]; this suggests that current recommendations that physicians discuss outdoor air pollution with cardiology patients [14], [17] may be effective in increasing activity change.

It is important to recognize that the results presented here are specific to those who changed activities on days with bad air quality; not merely those who were aware of or concerned about air quality. In order to change an activity due to bad air quality one must have planned an activity that could be changed on a day which had high ambient air pollution. Therefore the proportion of individuals who changed activities may be an underestimate of the proportion of individuals who would potentially have made changes if they had planned activities that were suitable to change. These results should be interpreted accordingly.

This study has a few limitations. The analysis relies on self-reported data for medical conditions. As a result, the prevalence of those with a respiratory or cardiovascular condition are likely to be underestimated, as not all specific respiratory or cardiovascular illnesses were included in the respiratory or cardiovascular condition variables, and it is possible that some individuals with these illnesses are undiagnosed. If this were a substantial factor in analysis, it would have served to weaken our ability to detect a difference in changing actions due to air quality between these groups. Recall bias related to reporting of activity change within the past 12 months may also be a concern. If recall bias were present in this analysis, it could have increased the strength of the association between being in a susceptible group and changing activity. Furthermore, given that this is a cross-sectional survey, we are unable to definitively establish that individuals were part of a susceptible group prior to changing activity due to bad air quality.

Local media may share information on local air quality alerts to their audience; however, the extent to which this was carried out during the study period may have varied considerably. Individuals may have also obtained information about local air quality directly from federal websites [8]. Wen and colleagues demonstrated that media alerts significantly contributed to an individual’s changing outdoor activity [21]. A limitation of the current analysis is that we were unable to assess the impact of the frequency and awareness of media alerts on individuals’ knowledge of air quality or change in activities, as these data were not collected within NHANES.

This study also has several strengths. It has a large sample size, which allows for comparisons among several demographic and susceptible groups. Additionally, NHANES is based on a representative sample of the United States population; therefore the proportions and odds ratios from this study are representative of the United States population.

### Conclusions

This analysis demonstrates that those with a respiratory condition are more likely to change activities based on poor air quality; however, more can be done by health and public health professionals to encourage persons susceptible to the effects of air pollution to make changes that will minimize their exposure to air pollution.

## Supporting Information

Figure S1 **Odds ratio (95% confidence interval) for changing activities, by susceptible group category.** NHANES = National Health and Nutrition Examination Survey; aOR = adjusted odds ratio; 95% CI  = 95% confidence interval. Probability of changing activity due to poor air quality by susceptible category, based on the adjusted regression model from Table 3 (main article); NHANES 2007–2010, N = 10,898. Model adjusted for gender, education, race/ethnicity, smoking status, and body mass index.
(JPG)

Table S1 **Self reported respiratory and cardiovascular conditions by activity change status, NHANES 2007–2010.**
(PDF)

Table S2 **Population distribution by type of activity changed, among those who changed at least one activity, NHANES 2007–2010, N = 1305.**
(PDF)

Table S3 **Odds ratio (95% confidence interval) for adjusted models predicting changing activity due to bad air quality, comparing the population with and without those who had no knowledge about air quality, NHANES 2007–2010.**
(PDF)

Table S4 **Odds ratio (95% confidence interval) for adjusted models predicting changing activities that are related to reducing exposure to or health impact from bad air quality, comparing the population with and without those who had no knowledge about air quality, NHANES 2007–2010.**
(PDF)
