# Supplementary figures and images for: Activity Change in Response to Bad Air Quality, National Health and Nutrition Examination Survey, 2007–2010
Source: PLoS One. 2012 Nov 30;7(11):e50526. doi: 10.1371/journal.pone.0050526 (PMC3511511; doi:10.1371/journal.pone.0050526)

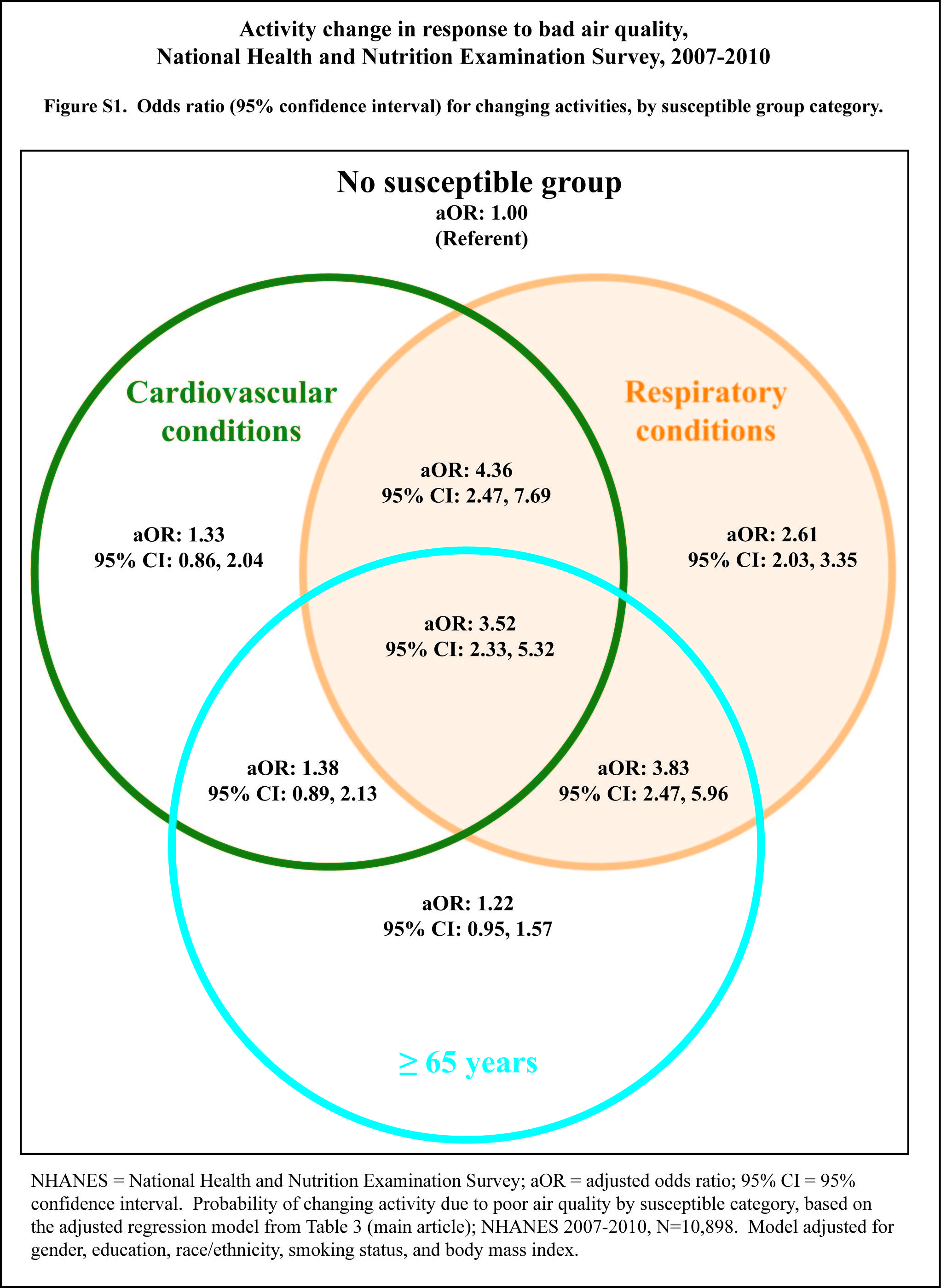

Supplement: Figure S1 — Odds ratio (95% confidence interval) for changing activities, by susceptible group category. NHANES = National Health and Nutrition Examination Survey; aOR = adjusted odds ratio; 95% CI = 95% confidence interval. Probability of changing activity due to poor air quality by susceptible category, based on the adjusted regression model from Table 3 (main article); NHANES 2007–2010, N = 10,898. Model adjusted for gender, education, race/ethnicity, smoking status, and body mass index. (JPG) [file pone.0050526.s001.jpg]
