# Supplementary material for: Activity Change in Response to Bad Air Quality, National Health and Nutrition Examination Survey, 2007–2010
Source: PLoS One. 2012 Nov 30;7(11):e50526. doi: 10.1371/journal.pone.0050526 (PMC3511511; doi:10.1371/journal.pone.0050526)
Supplement: Table S1 — Self reported respiratory and cardiovascular conditions by activity change status, NHANES 2007–2010. (PDF) [file pone.0050526.s002.pdf]

Activity change in response to bad air quality, National Health and Nutrition Examination Survey, 2007-2010

Table S1. Self reported respiratory and cardiovascular conditions by activity change status, NHANES 2007-2010.

		Changed activity		Did not change		Not informed	
**Characteristic**		**N[a]**	**Percent (95% CI)[b]**	**N[a]**	**Percent (95% CI)[b]**	**N[a]**	**Percent (95% CI)[b]**
Entire population		1305	12.0 (10.9, 13.1)	8895	81.5 (79.5, 83.5)	698	6.5 (4.5, 8.6)
Still have asthma[c]	Yes	224	25.0 (21.3, 28.6)	571	69.2 (64.7, 73.7)	44	5.8 (2.6, 9.0)
	No	1073	11.0 (9.9, 12.0)	8285	82.5 (80.4, 84.5)	651	6.6 (4.5, 8.6)
Emphysema[c]	Yes	64	27.0 (19.4, 34.6)	180	69.4 (61.7, 77.2)	14	3.5 (1.1, 6.0)
	No	1239	11.7 (10.6, 12.8)	8707	81.7 (79.7, 83.8)	681	6.6 (4.5, 8.6)
Still have chronic bronchitis[c]	Yes	87	30.1 (22.9, 37.3)	200	63.0 (55.9, 70.1)	19	6.9 (3.2, 10.7)
	No	1212	11.5 (10.5, 12.6)	8666	82.0 (79.9, 84.0)	677	6.5 (4.4, 8.5)
Congestive heart failure[c]	Yes	55	18.4 (11.4, 25.5)	243	72.3 (64.4, 80.2)	26	9.3 (3.8, 14.7)
	No	1247	11.9 (10.8, 13.0)	8624	81.7 (79.7, 83.7)	668	6.5 (4.4, 8.5)
Coronary heart disease	Yes	57	13.1 (9.2, 17.1)	360	80.5 (76.3, 84.7)	34	6.3 (2.6, 10.1)
	No	1,244	12.0 (10.8, 13.1)	8496	81.5 (79.4, 83.6)	661	6.5 (4.5, 8.6)
Angina[c]	Yes	54	19.5 (13.2, 25.7)	207	73.7 (67.1, 80.4)	18	6.8 (3.0, 10.6)
	No	1249	11.9 (10.7, 13.0)	8658	81.6 (79.6, 83.7)	677	6.5 (4.5, 8.5)
Heart attack[c]	Yes	84	17.9 (13.2, 22.6)	369	75.8 (70.4, 81.2)	31	6.3 (2.2, 10.3)
	No	1221	11.8 (10.7, 13.0)	8503	81.7 (79.6, 83.7)	666	6.5 (4.5, 8.6)
Stroke[c]	Yes	64	17.2 (12.4, 22.0)	309	77.5 (71.5, 83.5)	25	5.3 (2.1, 8.4)
	No	1240	11.9 (10.8, 13.0)	8567	81.6 (79.5, 83.6)	672	6.5 (4.5, 8.6)
Respiratory condition[c]	Yes	287	25.1 (21.7, 28.6)	775	69.1 (65.1, 73.0)	61	5.8 (3.1, 8.5)
	No	1018	10.6 (9.6, 11.6)	8120	82.8 (80.7, 85.0)	637	6.6 (4.5, 8.7)
Cardiovascular condition[c]	Yes	184	15.5 (12.2, 18.9)	924	78.0 (74.5, 81.4)	80	6.5 (3.7, 9.3)
	No	1121	11.7 (10.6, 12.8)	7971	81.8 (79.6, 84.0)	618	6.5 (4.5, 8.6)

NHANES = National Health and Nutrition Examination Survey; 95% CI = 95% confidence interval.

a. Unweighted sample N.

b. Percents are corrected for survey design, are row percents, and may not sum to 100 due to rounding.

c. Significant ($p<0.05$) Pearson's chi-squared test corrected for survey design, comparing the characteristic to activity change.
