# Supplementary material for: Activity Change in Response to Bad Air Quality, National Health and Nutrition Examination Survey, 2007–2010
Source: PLoS One. 2012 Nov 30;7(11):e50526. doi: 10.1371/journal.pone.0050526 (PMC3511511; doi:10.1371/journal.pone.0050526)
Supplement: Table S2 — Population distribution by type of activity changed, among those who changed at least one activity, NHANES 2007–2010, N = 1305. (PDF) [file pone.0050526.s003.pdf]

Activity change in response to bad air quality, National Health and Nutrition Examination Survey, 2007-2010

Table S2. Population distribution by type of activity changed, among those who changed at least one activity, NHANES 2007-2010, N=1305.

Activity classification	Activity	N[a]	Percent (95% CI)[a]
Possibly related to reduced exposure	Spent less time outdoors	919	69.4 (65.0, 73.8)
	Closed windows of your house	325	25.5 (20.7, 30.2)
	Canceled outdoor activities	195	13.4 (10.4, 16.3)
	Exercised indoors instead of outside	105	9.0 (6.9, 11.1)
	Wore a mask	114	9.3 (6.7, 11.8)
	Did less strenuous activities	162	12.7 (9.6, 15.9)
Possibly related to reduced health impact	Took medication	61	3.8 (2.5, 5.2)
Possibly related to reduced pollutant emissions	Drove your car less	141	11.2 (8.3, 14.1)
	Avoided roads with heavy traffic	83	5.5 (4.1, 7.0)
	Used buses, trains or subways	22	1.2 (0.3, 2.0)
Unclear impact	Used or changed air filter/air cleaner	45	4.2 (3.2, 5.3)
	Other change	81	5.4 (3.7, 7.1)
Reduced exposure or reduced health impact activity		1182	89.7 (87.4, 91.9)
Any activity		1305	100

NHANES = National Health and Nutrition Examination Survey; 95% CI = 95% confidence interval.

a. N is the unweighted sample N; percents are corrected for survey design, are row percents. Individuals could select more than one type of activity, therefore N's will not sum to 1305 and percents will not sum to 100.
