# Supplementary material for: Activity Change in Response to Bad Air Quality, National Health and Nutrition Examination Survey, 2007–2010
Source: PLoS One. 2012 Nov 30;7(11):e50526. doi: 10.1371/journal.pone.0050526 (PMC3511511; doi:10.1371/journal.pone.0050526)
Supplement: Table S3 — Odds ratio (95% confidence interval) for adjusted models predicting changing activity due to bad air quality, comparing the population with and without those who had no knowledge about air quality, NHANES 2007–2010. (PDF) [file pone.0050526.s004.pdf]

Activity change in response to bad air quality,
National Health and Nutrition Examination Survey, 2007-2010

Table S3. Odds ratio (95% confidence interval) for adjusted models predicting changing activity due to bad air quality, comparing the population with and without those who had no knowledge about air quality, NHANES 2007-2010.

Characteristic	Including not informed[a, b]	Excluding not informed[a]
N	10,898	10,200
Susceptible category (ref: none)		
Respiratory	2.61 (2.03, 3.35)	2.63 (2.04, 3.38)
Cardiovascular	1.33 (0.86, 2.04)	1.33 (0.86, 2.07)
≥ 65 years	1.22 (0.95, 1.57)	1.22 (0.95, 1.57)
Respiratory and cardiovascular	4.36 (2.47, 7.69)	4.37 (2.45, 7.80)
Respiratory and ≥ 65 years	3.83 (2.47, 5.96)	3.98 (2.52, 6.29)
Cardiovascular and ≥ 65 years	1.38 (0.89, 2.13)	1.39 (0.89, 2.15)
All three groups	3.52 (2.33, 5.32)	3.65 (2.35, 5.64)
Female (vs. male)	1.54 (1.34, 1.78)	1.56 (1.35, 1.81)
Education (ref: < high school)		
High school degree	1.46 (1.12, 1.90)	1.42 (1.10, 1.83)
Some college or 2-year degree	1.87 (1.49, 2.34)	1.87 (1.51, 2.33)
4-year degree or higher	2.27 (1.67, 3.09)	2.23 (1.64, 3.04)
Race/ethnicity (ref: non-Hispanic white)		
Non-Hispanic black	1.28 (0.93, 1.76)	1.20 (0.87, 1.66)
Hispanic	0.84 (0.61, 1.16)	0.84 (0.61, 1.17)
Other	1.22 (0.87, 1.70)	1.21 (0.86, 1.72)
Smoking status (ref: nonsmoker)		
Passive smoke exposure	0.88 (0.57, 1.35)	0.90 (0.58, 1.41)
Active smoker	0.90 (0.74, 1.09)	0.87 (0.72, 1.06)
Body mass index (ref: normal weight)		
Overweight	1.10 (0.89, 1.37)	1.11 (0.89, 1.39)
Obese	1.13 (0.96, 1.34)	1.14 (0.96, 1.35)

NHANES = National Health and Nutrition Examination Survey.

a. Odds ratios and confidence intervals account for survey design, weights, and non-response. Both models adjust for all variables shown.

b. This is the same as the adjusted model, Table 3 in the main article.
