# Supplementary material for: Activity Change in Response to Bad Air Quality, National Health and Nutrition Examination Survey, 2007–2010
Source: PLoS One. 2012 Nov 30;7(11):e50526. doi: 10.1371/journal.pone.0050526 (PMC3511511; doi:10.1371/journal.pone.0050526)
Supplement: Table S4 — Odds ratio (95% confidence interval) for adjusted models predicting changing activities that are related to reducing exposure to or health impact from bad air quality, comparing the population with and without those who had no knowledge about air quality, NHANES 2007–2010. (PDF) [file pone.0050526.s005.pdf]

**Activity change in response to bad air quality,**
**National Health and Nutrition Examination Survey, 2007-2010**

**Table S4. Odds ratio (95% confidence interval) for adjusted models predicting changing activities that are related to reducing exposure to or health impact from bad air quality, comparing the population with and without those who had no knowledge about air quality, NHANES 2007-2010.**

| Characteristic | Including not informed[a] | Excluding not informed[a] |
|---|---|---|
| N | 10,898 | 10,200 |
| Susceptible category (ref: none) | | |
| Respiratory | 2.79 (2.14, 3.62) | 2.80 (2.15, 3.64) |
| Cardiovascular | 1.38 (0.87, 2.20) | 1.39 (0.87, 2.23) |
| ≥ 65 years | 1.21 (0.91, 1.61) | 1.21 (0.91, 1.61) |
| Respiratory and cardiovascular | 4.75 (2.66, 8.47) | 4.75 (2.63, 8.59) |
| Respiratory and ≥ 65 years | 4.13 (2.68, 6.38) | 4.29 (2.74, 6.72) |
| Cardiovascular and ≥ 65 years | 1.55 (1.00, 2.42) | 1.56 (1.00, 2.44) |
| All three groups | 4.03 (2.65, 6.12) | 4.18 (2.69, 6.49) |
| Female (vs. male) | 1.65 (1.43, 1.92) | 1.67 (1.44, 1.95) |
| Education (ref: < high school) | | |
| High school degree | 1.39 (1.05, 1.85) | 1.35 (1.02, 1.77) |
| Some college or 2-year degree | 1.73 (1.37, 2.20) | 1.74 (1.38, 2.19) |
| 4-year degree or higher | 2.11 (1.51, 2.95) | 2.07 (1.48, 2.89) |
| Race/ethnicity (ref: non-Hispanic white) | | |
| Non-Hispanic black | 1.28 (0.92, 1.79) | 1.21 (0.86, 1.69) |
| Hispanic | 0.84 (0.60, 1.17) | 0.84 (0.60, 1.17) |
| Other | 1.19 (0.82, 1.72) | 1.18 (0.81, 1.73) |
| Smoking status (ref: nonsmoker) | | |
| Passive smoke exposure | 0.76 (0.47, 1.23) | 0.78 (0.48, 1.28) |
| Active smoker | 0.92 (0.74, 1.13) | 0.89 (0.72, 1.10) |
| Body mass index (ref: normal weight) | | |
| Overweight | 1.12 (0.92, 1.38) | 1.13 (0.92, 1.39) |
| Obese | 1.11 (0.94, 1.32) | 1.12 (0.94, 1.33) |

NHANES = National Health and Nutrition Examination Survey.

a. Odds ratios and confidence intervals account for survey design, weights, and non-response. Both models adjust for all variables shown.
